# Activating transcription factor 6 alleviates secondary brain injury by increasing cystathionine γ-lyase expression in a rat model of intracerebral hemorrhage

**DOI:** 10.18632/aging.205737

**Published:** 2024-04-10

**Authors:** Tianyu Liang, Sen Xu, Renyang Liu, Xiaoping Xia

**Affiliations:** 1 Emergency and Critical Care Center, Intensive Care Unit, Zhejiang Provincial People’s Hospital (Affiliated People’s Hospital, Hangzhou Medical College), Hangzhou 310014, Zhejiang, China; 2 Second Clinical Medical School, Zhejiang Chinese Medical University, Hangzhou 310053, Zhejiang, China; 3 Department of Intensive Care Unit, Taizhou Integrated Traditional Chinese and Western Medicine Hospital, Wenling, Zhejiang Province, China

**Keywords:** ATF6, CTH, intracerebral hemorrhage, neuroinflammation, cell death

## Abstract

Background: Intracerebral hemorrhage (ICH) comprises primary and secondary injuries, the latter of which induces increased inflammation and apoptosis and is more severe. Activating transcription factor 6 (ATF6) is a type-II transmembrane protein in the endoplasmic reticulum (ER). ATF6 target genes could improve ER homeostasis, which contributes to cryoprotection. Hence, we predict that ATF6 will have a protective effect on brain tissue after ICH.

Method: The ICH rat model was generated through autologous blood injection into the right basal ganglia, the expression of ATF6 after ICH was determined by WB and IF. The expression of ATF6 was effectively controlled by means of intervention, and a series of measures was used to detect cell death, neuroinflammation, brain edema, blood-brain barrier and other indicators after ICH. Finally, the effects on long-term neural function of rats were measured by behavioral means.

Result: ATF6 was significantly increased in the ICH-induced brain tissues. Further, ATF6 was found to modulate the expression of cystathionine γ-lyase (CTH) after ICH. Upregulation of ATF6 attenuated neuronal apoptosis and inflammation in ICH rats, along with mitigation of ICH-induced brain edema, blood-brain barrier deterioration, and cognitive behavior defects. Conversely, ATF6 genetic knockdown induced effects counter to those aforementioned.

Conclusions: This study thereby emphasizes the crucial role of ATF6 in secondary brain injury in response to ICH, indicating that ATF6 upregulation may potentially ameliorate ICH-induced secondary brain injury. Consequently, ATF6 could serve as a promising therapeutic target to alleviate clinical ICH-induced secondary brain injuries.

## INTRODUCTION

Intracerebral hemorrhage (ICH), a prevalent acute central nervous system (CNS) disease, is noted for its high mortality and morbidity rates [1, 2]. It accounts for roughly 15% of all stroke forms [3, 4]. Merely 20% of ICH patients retain functional independence six months after the ICH impairment [5]. Consequent to ICH, the brain incurs primary brain injury (PBI) and secondary brain injury (SBI) [6]. The former entails the formation and expansion of intracerebral hematoma, which inflicts direct mechanical damage to brain tissues [7]. The latter encompasses various pathophysiological processes, such as apoptosis activation, brain tissue ischemia and edema exacerbation around the hematoma, and related toxicities corresponding to these symptoms [8, 9]. Multiple studies underscore that neurological deterioration following ICH is mainly induced by secondary rather than primary brain injury [10]. Despite numerous research efforts and clinical trials to develop promising ICH therapies, the resultant mortality remains high, and no effective treatment has significantly improved patient prognosis [11]. Thus, comprehensively understanding the molecular mechanisms implicated in ICH-induced secondary brain injury is vital for effectively treating ICH [12].

Activating transcription factor 6 (ATF6) is a transcription factor regulated by endoplasmic reticulum (ER) stress, which instigates the expression of principal molecular chaperones within the ER [13, 14]. Intracellular environment disruptions, such as Ca^2+^ dysregulation, energy deficits, and abnormally increased protein synthesis, trigger unfolded protein accumulation in the ER, recognized as ER stress [15, 16]. This stress type induces the unfolded protein response (UPR), overseen by at least three distinct branches, inclusive of protein kinase R-like ER kinase and inositol-requiring enzyme 1 (IRE1) [17]. Evidence points to a consistent over-expression of ATF6 in intracerebral hematoma, suggesting a significant association between ATF6 and stroke [18]. Furthermore, ATF6 assumes a crucial role in cellular apoptosis, inflammatory responses, oxidative stress, and other aspects [19–22].

Hydrogen sulfide (H2S) modulates a plethora of physiological and pathological effects through numerous regulatory forms and signaling pathways [23]. Predominantly generated by three enzymes—cystathionine β-synthase (CBS), cystathionine γ-lyase (CTH), and 3-mercaptopyruvate sulfur transferase (3-MST) [24]—in the brain, CTH is primarily localized within the brain’s micro vessels. The CTH activity in the human brain is 100 times higher than that in the mouse brain, indicating its significant role in the human central nervous system [25]. According to existing research, ATF6 can foster autophagy by elevating CTH levels, thereby aiding neurological damage repair and behavioral improvement. Consequently, it is plausible to speculate that ATF6 may regulate CTH expression via certain pathways, thus attaining the effect of mitigating brain injury.

Collectively, ATF6 has been linked to neuronal survival, apoptosis, and neurite outgrowth across a spectrum of central nervous system (CNS) disorders. In instances of brain damage, ATF6 may assume a protective role through apoptosis inhibition. It is conjectured that a correlation may exist between ATF6 and CTH within ICH-induced brain tissues. However, the expression levels of ATF6 in rats exhibiting ICH impairment have not been examined in previous studies. Consequently, this study explores the spatio-temporal expression levels of ATF6 induced by ICH, and discusses their roles in ICH-induced secondary brain injury (SBI).

**Figure 1:**
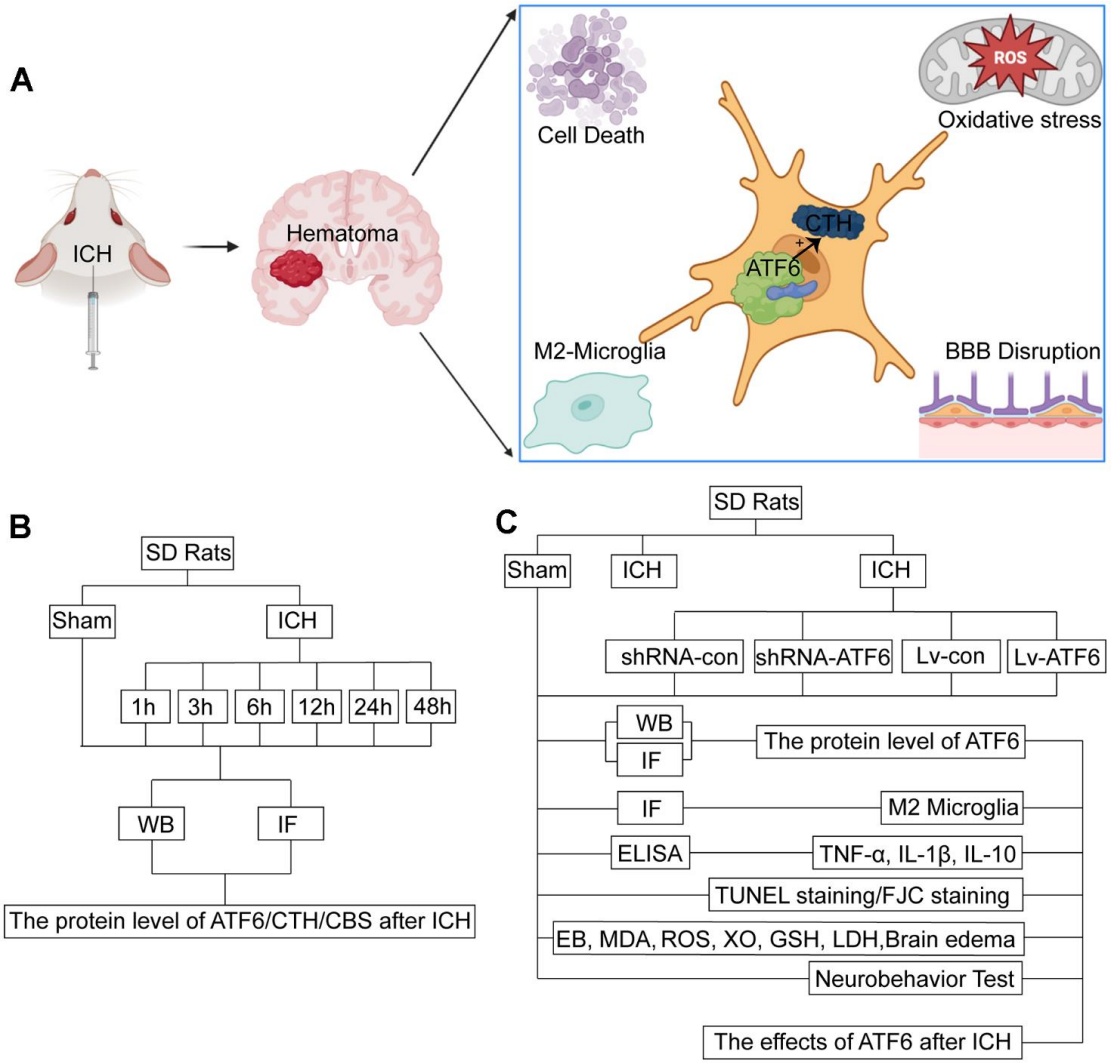
**Experimental mechanism diagram and experiment design.** (**A**) Experimental mechanism: after ICH, ATF6 in neurons can promote the transformation of microglia into M2 by regulating the expression of CTH, inhibit inflammation, inhibit cell death, protect the blood-brain barrier, and relieve oxidative stress. (**B**) Experiment 1: the time course changes of ATF6 after ICH. (**C**) Experiment 2: the role of ATF6 in ICH-induced brain injury.

In conclusion, ATF6 has a strong protective effect on the posterior brain tissue of ICH (Figure 1A), and ATF6 is expected to become a target for clinical treatment of ICH, providing a new treatment option for ICH patients.

## MATERIALS AND METHODS

### Animals and ethical considerations

All experiments were approved by the Institutional Animal Care Committee of the Zhejiang Provincial People’s Hospital and conducted in adherence to the National Institutes of Health’s Guidelines on Animal Care and Use. Adult male Sprague-Dawley (SD) rats, weighing between 280-300g, were procured from the Animal Center of the Chinese Academy of Sciences, Shanghai, China. Following the Guide for the Care and Use of Laboratory Animals of the National Institutes of Health and abiding by the ARRIVE (Animal Research: Reporting of *In Vivo* Experiments) guidelines, adult male Sprague-Dawley (SD) rats (weight 280-320g; Animal Center of Chinese Academy of Sciences, Shanghai, China) were accommodated in an environment with a temperature of 18-26° C, humidity of 40-70%, noise below 85 decibels, ammonia concentration below 20PPm, and ventilation of 8-12 times/hour under a standard 12-hour light/dark cycle. Food and water were supplied without restriction. Anyway, we strived to minimize the number of animals used and the pain of the experimental process.

### Experimental grouping

The experiments were divided into two main sections, namely Experiment 1 and Experiment 2. Rats were assigned to different groups using an equivalent random assignment method, which involved a researcher, blind to the groupings, assigning the rats using a random-number table.

In Experiment 1 (Figure 1B), following the induction of ICH, the 36 surviving ICH rats were divided into several discrete ICH groups. Experiment 1 was comprised of the following seven groups: a Sham group and six experimental groups corresponding to the time after ICH: 1, 3, 6, 12, 24, and 48 hours under the influence of ICH (each group included six subjects randomly selected from the surviving rats). After the ICH treatment, the rats were subjected to deep anesthesia using sodium pentobarbital at appropriate intervals, and relevant cerebral tissues were collected for further investigation. For each subject, two cerebral coronal slices were procured: one slice 3 mm anterior and the other 4 mm posterior to the point of coronal injection. The right basal ganglia were promptly isolated from the 3 mm thick sections for the Western blot assay. Tissue from the 4 mm thick slice was fixed in 4% paraformaldehyde, embedded in paraffin, and then sectioned into 4 μm slices for immunofluorescence analyses.

Experiment 2 was made up of the following six groups, each containing 30 rats randomly selected from the previously mentioned Sham and ICH subjects: the Sham group, ICH group, ICH+ shRNA-control group, ICH+shRNA-ATF6 group, ICH+LV-control group, and ICH+LV-ATF6 group (Figure 1C).

12 rats were utilized to examine behavioral impairments using the Morris water maze and rotarod performance tests. 72 hours after ICH, 6 rats were promptly euthanized to collect cerebral tissue, blood, and cerebrospinal fluid (CSF) samples for Western blotting, immunofluorescence assays, ELISA, terminal deoxynucleotidyl transferase-mediated dUTP nick-end labeling (TUNEL), fluoro-jade C (FJC) staining, reactive oxygen species (ROS) and Lactate Dehydrogenase (LDH) analyses. The remaining 6 subjects in each group were chosen for the evaluation of brain edema at 72 hours after ICH. To ensure impartial analysis of behavioral damage and cerebral edema, the experimenters remained completely unaware of the group assignments.

### The establishment of ICH model *in vivo*

The *in vivo* ICH model was established using an autologous whole-blood model as detailed in previous research [26]. Briefly, Sprague-Dawley rats were anesthetized with isoflurane (induction concentration was 3%, maintenance concentration was 2%) and secured to a stereotaxic apparatus (ZH-Lanxing B-type stereotaxic frame, Anhui, China). Then, 70 μL of autologous blood was slowly administered unilaterally (20 μL/min) to the right basal ganglia using a microliter syringe. The injection site was 3.5 mm lateral to the midline, 0.2 mm posterior to the bregma, and 5.5 mm ventral to the cortical surface. To prevent reflux, the needle was left in place for an additional 5 minutes post-injection. The blood that actually entered the rat brain tissue was about 70 ul. This is equivalent to 50-60 ml of human intracerebral hemorrhage. The survival rate of ICH rats induced by this method can reach more than 80% within one month. For sham-treated rats, 70 μl of physiological saline was administered intracerebrally. Following this, bone wax was used to seal the burr hole, and the skin incision was disinfected and sutured. Throughout the surgery, the rats were placed supine on a heating blanket to maintain body temperature within approximately 37 ± 0.5° C.

To ensure the uniformity of the amount of hematoma in the brain parenchyma, we spent a lot of time and animals in our previous model construction, and after we became familiar with the ICH model construction, we started the formal experiment. We just randomly selected one ICH rat and made brain slices to observe the location and size of the hematoma. Some of the hematoma in the selected rat entered the ventricle, but most of the hematoma was in the basal ganglia. It is necessary to accurately locate the basal ganglia area by stereotactic machine and inject blood as slowly as possible to ensure the homogeneity of the hemorrhage location and hematoma. Clinically, there are also many patients with cerebral hemorrhage breaking into the ventricles. Although we try to simulate the basal ganglia cerebral hemorrhage, intraventricular hemorrhage is inevitable in the process of modeling. Based on the rigor of basic experiments and control methods, ICH may have a certain effect on the behavior of rats, but the probability of the effect on each group of rats is the same, so we can only use a stable technique as far as possible, as accurate as possible positioning, as slow as possible to eliminate this effect. In short, we must ensure the stability of modeling technology.

### Lentiviral transduction

In line with previous literature [27], we performed intracerebroventricular injection of lentivirus seven days before ICH induction in rats. Briefly, we anesthetized the Sprague-Dawley rats and secured them in a stereotaxic apparatus, after which we drilled a left-sided hole 1.5 mm lateral and 1.1 mm posterior to the bregma. We then slowly injected a 5 μl dose of lentivirus into the lateral ventricle at a rate of 0.5 μl/min to a depth of 3.5 mm and left the needle in place for 5 minutes.

### Western blot assay

In agreement with previous studies [28], we used the Western blot assay to measure protein levels. Brain samples, procured from the ipsilateral side of the brain 3 mm anterior to the needle point, were used for Western blotting. We selected ICH rat brain tissue at the time point when ATF6 expression peaked, signifying maximum ATF6 function, for subsequent experiments. We homogenized the extracted right basal ganglia from each rat and centrifuged the samples at 12,000 g at 4° C for 10 minutes, followed by supernatant collection. Protein concentrations were determined using the Enhanced BCA Protein Assay Kit (Beyotime Biotechnology, Shanghai, China). Equivalent proteins (30 μg/lane) were subjected to SDS polyacrylamide gel electrophoresis, followed by electrotransfer to a polyvinylidene difluoride membrane (Millipore, USA). The membranes were blocked with 5% nonfat milk for 1 hour and incubated with ATF6 primary antibodies (concentration 1:200, ab37149, Abcam, UK)/CTH primary antibodies (concentration 1:50, ab136604, Abcam)/CBS primary antibodies (concentration 1:500, ab313382, Abcam) overnight at 4° C. We used anti-ATF6 and anti-β-tubulin (concentration 1:100, ab18207, Abcam) antibodies for immunoblotting. The membranes were then washed three times (5 min/wash) with PBST (PBS + 0.1% Tween 20) and incubated with horseradish peroxidase (HRP)-linked secondary antibodies (Santa Cruz Biotechnology, USA). We used the enhanced chemiluminescence kit (Affinity, Jiangsu, China) for signal detection. The relative protein quantity was calculated using ImageJ software (National Institutes of Health, USA) by a researcher unaware of the rat group assignments.

### Immunofluorescence staining

As previously detailed [29], the brain samples obtained from the brain tissue 4 mm after the insertion point were cut into equal 4 μm sections to perform staining. We fixed the brain samples in 4% paraformaldehyde at 4° C overnight, embedded them in paraffin, and sliced them into 4 μm-thick sections. After antigen retrieval and blocking, the sections were incubated with primary antibodies for ATF6 (concentration 1:100, ab37149, Abcam) and NeuN (concentration 1:100, ab177487, Abcam) overnight at 4° C, followed by triple washing in TBST (PBS+0.1% Tween 20). Subsequently, the appropriate secondary antibodies were applied to the sections at 37° C for 1 hour, followed by triple washing in PBST. Ultimately, we cover-slipped the slices with anti-fading mounting medium that included 4,6-diamino-2-phenyl indole (DAPI, SouthernBiotech, USA). We observed at least six microscopic fields with 400x magnification in the cortex closest to the hematoma in the ipsilateral cerebral hemisphere in each slice. A researcher, blind to the rat grouping, independently obtained representative photos from at least six tests with six different rats. We used ImageJ software (National Institutes of Health, USA) to analyze the relevant fluorescence intensity and count positive cells.

### ELISA

At 24 hours after ICH (immediately prior to euthanasia), we collected blood samples from all subjects via heart puncture and CSF samples from the foramen magnum. We prepared all blood samples through centrifugation at 1000g for 5 minutes at 4° C. We subjected CSF samples to immediate centrifugation at 12000g for 30 minutes at 4° C. Next, we gathered the supernatants for analysis of IL-1β, TNF-α, and IL-10 expressions using specific ELISA kits (Bio-Swamp, Wuhan, China), following the manufacturer’s guidelines.

### ROS analysis

We measured cerebral ROS levels, indicative of oxidative stress, using an ROS assay kit (Beyotime Biotechnology, China). We prepared brain tissue samples through homogenization and centrifugation at 12000g for 10 minutes at 4° C, and collected the supernatants. ROS levels were measured using the oxidant-sensitive probe 2,7-dichlorofluorescein diacetate (DCF-DA). We then detected fluorescent intensity using a fluorometric microplate reader (Molecular Devices, USA) with excitation at 485 nm and emission at 530 nm. ROS concentrations across groups were denoted as fluorescent intensity per total protein mass (mg). We normalized all sample ROS levels to those in the Sham group.

### Glutathione level measurement

We measured GSH levels using the Ellman method, in which GSH reacts with 5,5-dithiobis-2-nitrobenzoic acid to form a product with maximum absorbance at 410 nm. We express the results as nanomoles per gram of wet tissue.

### Xanthine oxidase (XO) activity measurement

We determined XO activity spectrophotometrically following the Prajda and Weber method, which relies on the conversion of xanthine to uric acid, characterized by increased absorbance at 292 nm (eM 9.2 · 103). We define one unit of activity as 1 μmol of uric acid formed per minute and express the data as U/mg protein.

### Evans blue

We injected six rats in each group with 4 ml/kg of Evans blue solution (MKBQ3656V, Sigma-Aldrich, Merck KGaA, Darmstadt, Germany) via the caudal vein. After 1 hour of systemic circulation, we anesthetized the rats and perfused them with normal saline to remove the colorless liquid. We collected the injured brain tissue, weighed it, and cut it into pieces. We then dissolved the tissue in 1 ml 50% trichloroacetic acid at 60° C, incubated it for 24 hours, and centrifuged it at 1000 × g for 10 minutes. We diluted the supernatant using absolute ethanol and detected the absorbance value at the wavelength of 620 nm. We calculated the content of Evans blue (μg/g) in the corresponding brain tissue.

### MDA analysis

Lipid peroxidation in hippocampal samples was evaluated using an MDA assay kit (S0131; Beyotime Biotechnology) to quantify MDA concentration. We weighed each hippocampus before creating a homogenate with PBS on ice (10% hippocampal-tissue weight) which was then centrifuged at 12,000 g for 10 minutes at 4° C to obtain the supernatant. We added assay reagents to the mixtures, heated them at 100° C for 15 minutes, and after cooling to room temperature, we centrifuged them at 1000 g for 10 minutes to obtain the supernatant. We determined MDA concentration by measuring the absorbance at 532 nm.

### The quantity of brain water content

Following previous methods [30], we employed the wet-dry technique to determine cerebral edema. Briefly, 24 hours after ICH induction, we injected subjects with sodium pentobarbital (40mg/kg) intraperitoneally and promptly extracted the intact brain. We split each brain along the midline into two hemispheres, which were further divided into five sub-regions: ipsilateral basal ganglia (Ipsi-BG), ipsilateral cortex (Ipsi-CX), contralateral basal ganglia (Cont-BG), contralateral cortex (Cont-CX), and cerebellum (CB). We immediately weighed these sections to record their wet weights. After dehydrating the samples at 100° C for 72 hours, we recorded their dry weights. We calculated the water content proportion as follows: [(wet weight - dry weight) / wet weight] x 100%.

### Neurobehavioral evaluations

All rats underwent evaluations encompassing seven distinct areas: body proprioception (BP), climbing (CL), forelimb outstretching (FO), limb symmetry (LS), lateral turning (LT), spontaneous activity (SA), and vibrissae touch (VT), as previously described [31]. Further details about these behavioral tests are provided in Table 1. Rats were trained three times per day for three days before the ICH model was established. The average duration of the test prior to ICH onset was recorded as the baseline value (Pre). The test was carried out on days 1, 3, 7, 10, 14, 21, 28, and 35 after ICH because these time points are the peak of the development of pathological events such as oxidative stress, brain edema, and neuroinflammation in the acute period after ICH, and are the key time points for the recovery of nerve function after ICH.

**Table 1:** Neurobehavioral evaluation: neuroscore scoring criteria for the sub-tests.

**Category**	**Behavior**	**Score**
Spontaneous Activity (SA)	Animal was akinesitic	0
	Animal moves slowly or minimally	1
	Animal approached 1-2 walls	2
	Animal approached at least 3 walls of the cage or raised on hindlimbs to explore the top of the cage	3
Vibrissae Proprioception (VP)	-	0
	Animal had a unilateral response	1
	Animal had either a weak bilateral response or weak left response and brisk right response	2
	Animal had a brisk bilateral response	3
Axial Sensation (AS)	-	0
	Animal had no response on left side	1
	Animal had either a weak bilateral response or weak left response and brisk right response	2
	Animal had a brisk bilateral response	3
Limb Symmetry (LS)	Hemiparesis	0
	Left forelimb or left hindlimb flexed	1
	Asymmetric extension	2
	All limbs were extended symmetrically	3
Lateral Turning (LT)	Animal had no turning at all on one side	0
	Animal had unequal turning	1
	Animal turned bilaterally less than 45° on both sides	2
	Animal turned bilaterally at least 45° on both sides	3
Forelimb Walking (FW)	Animal had a paretic forelimb	0
	Animal walked in circles	1
	Animal walked asymmetrically or to one side	2
	Animal briskly walked symmetrically on forepaws	3
Climbing (CL)	-	0
	Animal failed to climb or circled instead of climbing	1
	Animal climbed to the top and had a weak grip or animal climbed but had a strong grip	2
	Animal climbed to the top and had a strong grip	3

### TUNEL staining

TUNEL staining was utilized to detect apoptosis in brain tissues [32]. Initially, brain slices were dehydrated at 70° C for an hour. We then gradually deparaffinized the sections using 100% dimethylbenzene, followed by treatment with a series of ethanol solutions (100% twice, 95% twice, and 80%). We subsequently treated these samples with TUNEL staining reagents for an hour at 37° C. Finally, the TUNEL-stained slices were incubated with the NeuN antibody at 4° C overnight to identify neuronal apoptosis. An experimenter, blind to the rat groupings, used a fluorescent microscope (Nikon, Tokyo, Japan) to visualize TUNEL-positive neurons. The apoptotic index, defined as the proportion of TUNEL-positive cells in the brain, was used to quantify brain tissue apoptosis.

### FJC staining

Neuronal degeneration was assessed via FJC staining as per existing studies [33]. Briefly, brain slices were deparaffinized and rehydrated, immersed in 0.06% potassium permanganate solution (Sigma-Aldrich, USA) for 15 minutes, rinsed in deionized water, and then soaked in FJC working solution containing 0.1% acetic acid for 30 minutes. These slices were subsequently dehydrated in an incubator, rinsed with 100% dimethylbenzene for 2 minutes, and cover-slipped with neutral balsam. A researcher, unaware of the rat groupings, visualized and counted FJC-positive cells under a fluorescent microscope (Nikon, Japan).

### Morris water maze tests

We evaluated cognitive capacity via the Morris water maze tests, as described in previous research [34]. The water maze was divided into four quadrants with a target platform hidden 2 cm below the water surface in the center of the third quadrant. The water was maintained at a temperature of 25±2° C and dyed with a special black pigment. Consistent visual reference points were maintained around the water maze throughout the training and testing processes. The rats were placed in the maze at each quadrant and given 60 seconds to find the target platform. If a rat failed to reach the target platform within 60 seconds, they were guided using a stick. Upon reaching the platform, the rats were allowed to stay for 10 seconds to reinforce the associated learning and memory. This training process was repeated over five consecutive days, with ICH induction occurring the day after training. Escape latency (EL), the time taken from the start position to the platform, was recorded. Rats were placed in the first quadrant and allowed to find the target platform. On days 30, 31, 32, 33 and 34 after ICH, ELs and swimming distances were tested and documented. We used the mean EL and distance to evaluate each rat’s learning ability and cognitive function. After 5 days of water maze testing, in order to judge the spatial memory ability of the rats for the target platform, we conducted a special water maze experiment on the rats on the 35 days after ICH modeling. After the platform was removed and the rats were released, the number of times the rats crossed the area where the platform was located was recorded, and the time the rats stayed in the target quadrant was recorded, which would serve as a reference indicator of the memory function of the rats.

### Rotarod performance test

The rotarod test primarily assessed the effects of therapeutic intervention on vestibulomotor function. In brief, rats were placed on a rotarod cylinder (ZH-300B, Anhui Zhenghua Biological Equipment Co. Ltd., China), and the duration of each rat remaining on the cylinder was recorded. The cylinder’s speed was consistently increased from 4 to 30 rpm within one minute. The experiment concluded when the rat either fell off or gripped the cylinder, making two consecutive revolutions. Each rat underwent training three times per day for three days before establishing the ICH model. The average duration on the rotarod before ICH induction was recorded as the baseline value (Pre). Subsequently, tests were conducted on days 1, 3, 7, 10, 14, 21, 28, and 35 after ICH to evaluate early impacts on motor function.

### Foot fault test

We conducted the foot fault test, as described in prior research, to evaluate sensorimotor coordination during spontaneous locomotion [35]. Rats were recorded for 60 seconds from beneath a grid. A blinded investigator analyzed these recordings, accounting for the number of total steps and foot faults made by the left limbs (impaired side, contralateral to the lesion). Foot faults were identified when the rat misplaced its impaired forepaw or hind paw, causing the paw to fall through the grid. The obtained data were presented as percentages of foot faults over total steps taken by that paw. Rats were trained three times per day for three days before the ICH model was established. The average duration of the test prior to ICH onset was recorded as the baseline value (Pre). Similar to the rotarod test, the foot fault tests were carried out on days 1, 3, 7, 10, 14, 21, 28, and 35 after ICH.

### Adhesive removal test

As mentioned in the previous literature, we used adhesive removal test to measure the sensory function of the affected opposite limb in rats [36]. Rats were trained three times per day for three days before the ICH model was established. The average duration of the test prior to ICH onset was recorded as the baseline value (Pre). Similar to the rotarod test, the foot fault tests were carried out on days 1, 3, 7, 10, 14, 21, 28, and 35 after ICH.

### Statistical analysis

All results are expressed as mean ± standard deviation (SD). We used GraphPad Prism 6.0 (GraphPad Software, USA) for statistical analysis. The Kolmogorov-Smirnov test analyzed the normal distribution of data sets. For two normally distributed groups, we applied a two-sided unpaired Student’s t-test for comparison, and the Mann-Whitney U test for non-parametric results. We utilized repeated measures ANOVA to analyze data from the Morris water maze. Parameters with a *p* < 0.05 were considered statistically significant. In this study, power analysis determined sample sizes for the animal ethics dossier application, and each two-sided unpaired Student’s t-test or Mann-Whitney U test was conducted using SAS 9.0 (SAS Institute Inc., USA). All power analyses exceeded 0.8 in the current study.

### Availability of data and materials

The datasets used and/or analyzed during the present study are available from the corresponding author on reasonable request.

## RESULTS

### General observation

Our study involved various experimental ICH groups, wherein we measured parameters such as body weight, body temperature, blood pressure, arterial blood gases, and glucose levels. Notably, there were no significant differences among the groups. A total of 204 experimental rats were used, including 30 in the Sham group and 174 in the ICH group. In the Sham group, no mortality was observed, while in the ICH group, we recorded a mortality rate of 10.3%, which was consistent with the animal mortality rates reported in existing study [37]. 18 rats died from ICH modeling, and we speculated that the death might be caused by: 1. The speed of blood injection was not balanced, and occasionally the speed was too fast, which led to the increase of intracranial pressure, and finally led to the death of the rats. 2. Errors in stereotaxic orientation caused blood to enter the cerebral ventricle and other areas, accelerating the death of rats. 72 rats undergoing behavioral experiments were euthanized. The mortality rate did not differ significantly across the experimental groups. In Experiment 2, injection of interfering reagents had no significant impact on the mortality rate of ICH rats (Table 2). We monitored the health and behavior of the animals every 1h for 24 hours after molding, and at a frequency of 12h after 24 hours. This aligns with the approved ICH model mortality rate for Sprague-Dawley (SD) rats, as per the National Ethical Committee in Animal Experimentation (CEEA, Comiteì d’Ethique en Experimentation Animale) of the French Ministry for Education and Research.

**Table 2:** Mortality in experimental group.

**Group**	**Total**	**Death number**	**Survival number**	**Mortality rate**	**Death time (after ICH)**
ICH-1h	6	0	6	0	-
ICH-3h	7	1	6	14.3%	1.5h
ICH-6h	7	1	6	14.3	4h
ICH-12h	8	2	6	25%	2h and 3.5h
ICH-24h	7	1	6	14.3%	1h
ICH-48h	6	0	6	0	5h
ICH	27	3	24	11.1%	2.5h, 4.5h and 7h
ICH+ shRNA-con	26	2	24	7.7%	3.5h and 5h
ICH+shRNA-ATF6	26	2	24	7.7%	3h, 5.5h and 6h
ICH+ Lv-con	28	4	24	14.2%	1h, 3.5h, 4h and 7h
ICH+Lv-ATF-6	26	2	24	7.7%	4.5h and 6.5h

### ATF6 protein level was significantly up-regulated in the ICH-induced tissues of brain

**Figure 2:**
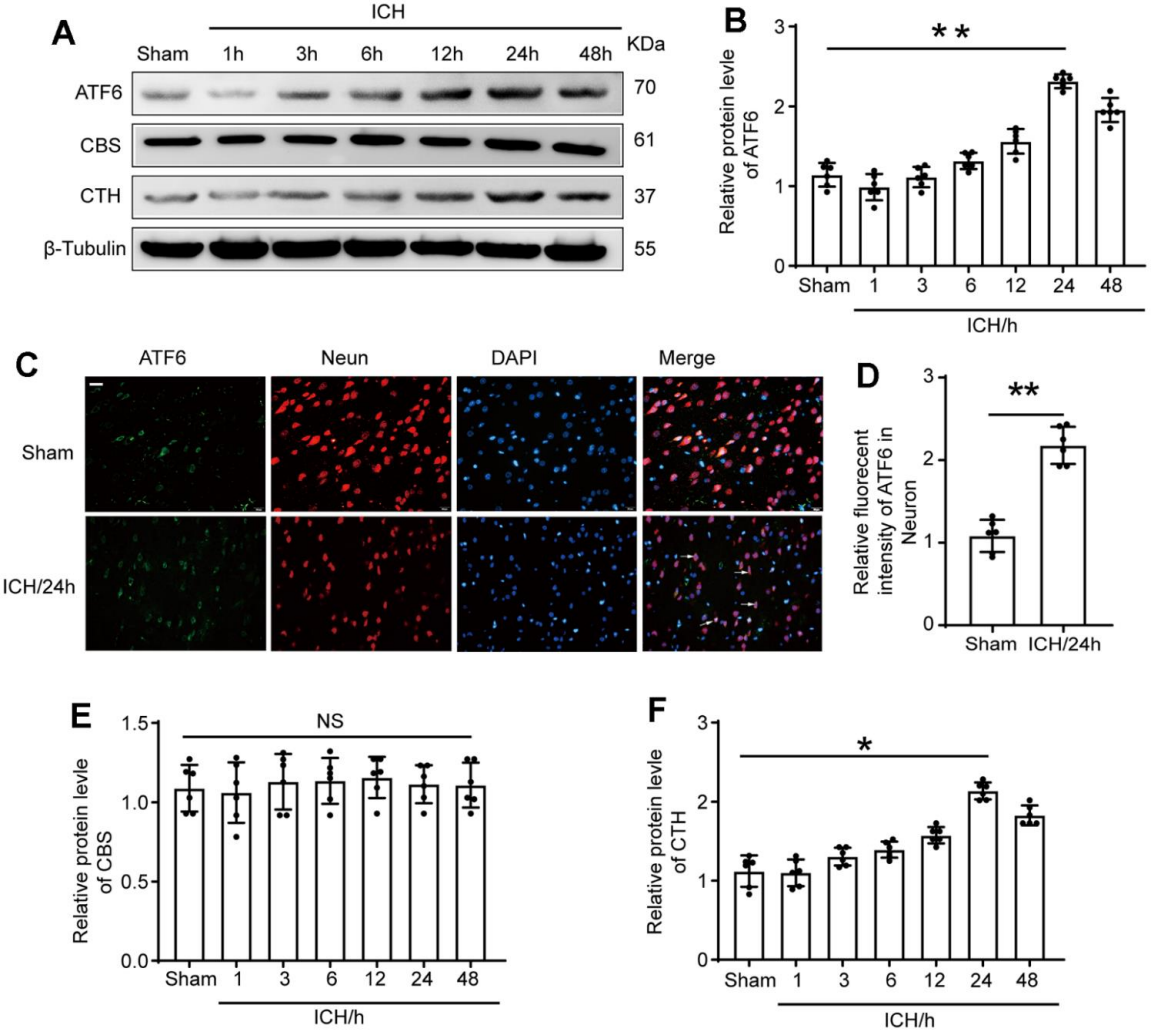
**The protein level of ATF6 in neuron increased significantly at 24 h after ICH.** (**A**, **B**) Western blot analysis and quantification of ATF6 at 1h, 3h, 6h, 12h, 24h and 48h after ICH. (**C**, **D**) Double immunofluorescence analysis of ATF6 (green) and neuron (red) in brain sections. Nuclei were labeled with DAPI (blue). Arrow indicated ATF6 positive cells. Scale bar = 50 μm. (**E**, **F**) Western blot analysis quantification of CBS and CTH at 1h, 3h, 6h, 12h, 24h and 48h after ICH. The black dots represent individual data in each group. **p < 0.01 and *p < 0.05 vs. sham group, NS, no significant difference vs. Sham group, n = 6.

We aimed to elucidate the protein expression levels of ATF6, CBS, and CTH in brain tissues following ICH at varying time intervals. For this purpose, we performed Western blotting assays. We found a substantial increase in ATF6 protein expression in ICH-induced tissues compared to the Sham group. In response to ICH, ATF6 levels started to rise, peaking at 24 hours and remaining elevated up to 48 hours after ICH. Concurrently, CTH expression varied in parallel with ATF6 changes, whereas CBS levels remained constant, unaffected by ICH (Figure 2A, 2B, 2E, 2F). We further identified that ATF6 was predominantly expressed in neurons through double-immunofluorescent staining (Figure 2C, 2D).

### Lentiviral transduction increased and decreased the expression of ATF6 protein level in brain tissues respectively

**Figure 3:**
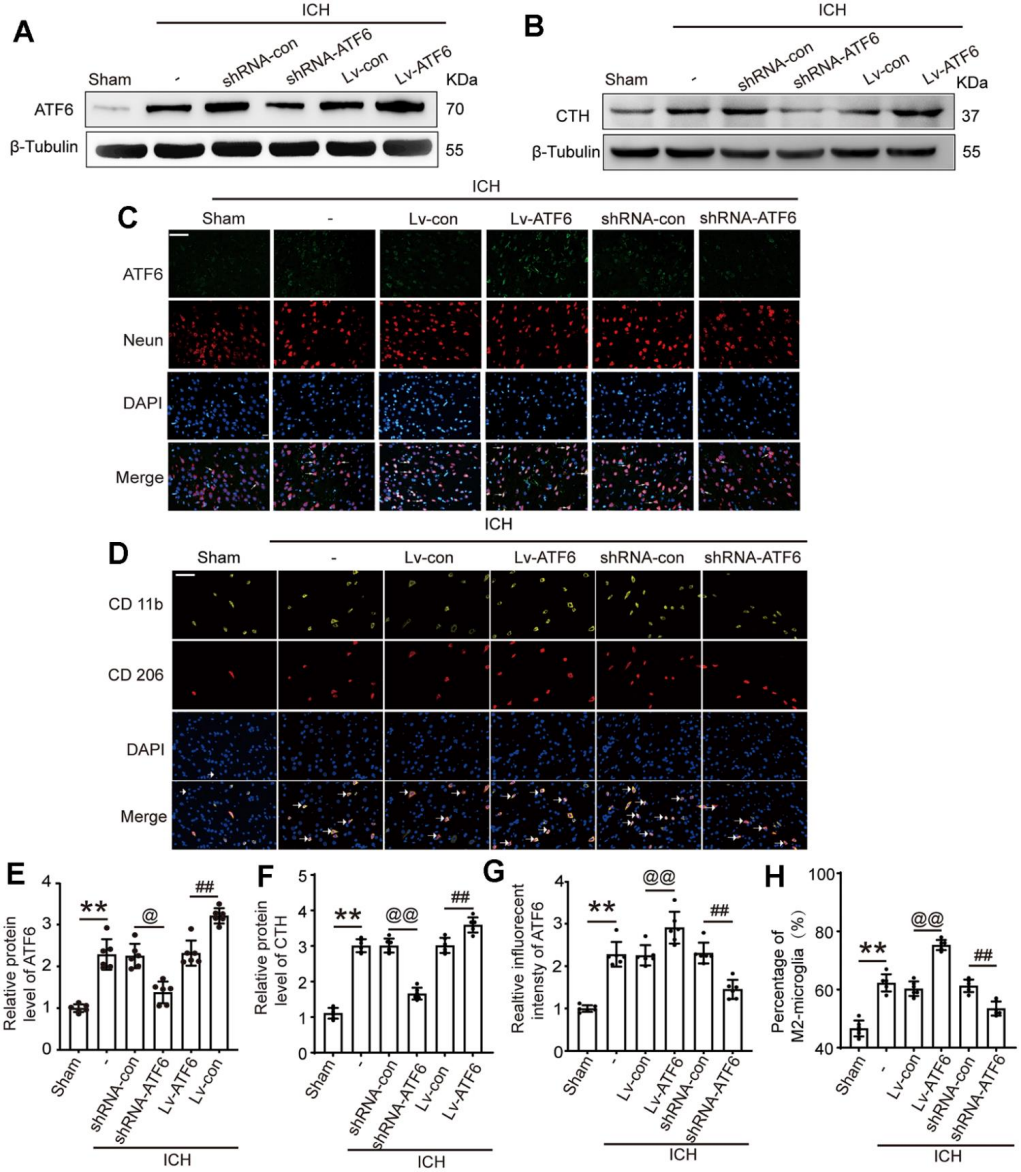
**Intervention efficiency of overexpression and interfering RNA lentivirus on ATF6.** (**A**) Representative Western blot image of ATF6 in intervention groups. (**B**) Representative Western blot image of CTH in intervention groups. (**C**) Double immunofluorescence analysis of ATF6 (green) and Neuron (red) in brain, arrow indicated ATF6 positive cells. Nuclei were labeled with DAPI (blue). (**D**) Double immunofluorescence analysis of microglia (yellow) and M2-microglia (red) in brain, arrow indicated M2-microglia positive cells. (**E**) Western blot analysis quantification of ATF6 in intervention groups. (**F**) Western blot analysis and quantification of CTH in intervention groups. (**G**) The relative fluorescent intensity of ATF6 in neuron cells. (**H**) The percentage of M2-positive microglia was analyzed. Scale bar = 50 μm. The black dots represent individual data in each group. **p < 0.01 and *p < 0.05, ^@@^*p* < 0.01 and ^@^*p* < 0.05, ^##^*p* < 0.01 and ^#^*p* < 0.05, n = 6.

To ascertain the role of ATF6 in ICH-induced brain damage, we manipulated ATF6 expression via lentiviral transfection. Figure 3A, 3E depict that the shRNA-ATF6 group had significantly lower ATF6 protein levels compared to the shRNA control group. In contrast, the Lv-ATF6 overexpression group showed a marked increase in ATF6 protein levels. Similarly, the expression level of CTH was significantly decreased in the shRNA-ATF6 group, the results indicated that the change of ATF6 expression level affected the expression of CTH (Figure 3B, 3F). The results of immunofluorescence experiment showed that compared with shRNA control group, the expression of ATF6 in shRNA-ATF6 group was significantly decreased, while the expression of ATF6 in Lv-ATF6 group was significantly increased (Figure 3C, 3G). As various studies have noted, ATF6 is a crucial regulator of the inflammatory response. Through immunofluorescence (IF) experiments, we monitored phenotypic changes in Microglia. Our findings revealed that an increase in ATF6 expression correlated with a rise in the proportion of M2 type Microglia, whereas a decrease in ATF6 led to a reduction in M2 type Microglia. These findings suggest that ATF6 could foster the transformation of Microglia to the M2 type, thereby inhibiting the inflammatory reaction (Figure 3D, 3H).

### Elevated ATF6 protein levels inhibit central system inflammatory response and BBB impairment

**Figure 4:**
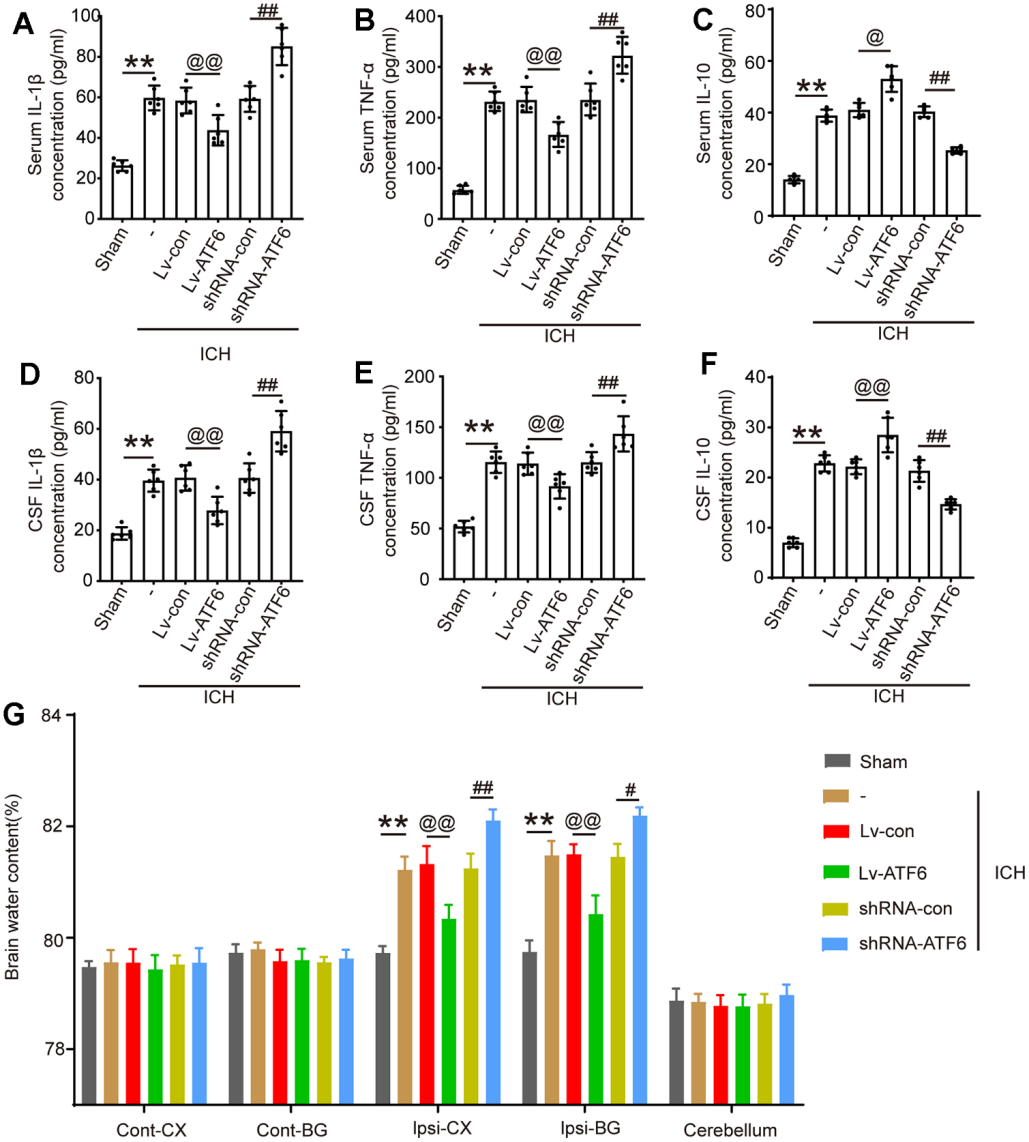
**ATF alleviates the inflammatory response and brain edema after ICH.** (**A**) IL-1β in serum. (**B**) TNF-α in serum. (**C**) IL-10 in serum. (**D**) IL-1β in CSF. (**E**) TNF-α in CSF. (**F**) IL-10 in CSF. (**G**) Brain water content. The black dots represent individual data in each group. **p < 0.01 and *p < 0.05, ^@@^*p* < 0.01 and ^@^*p* < 0.05, ^##^*p* < 0.01 and ^#^*p* < 0.05, n = 6.

As IF in Figure 3D showed that when the expression level of ATF increased, the percentage of M2-microglia increased. In alignment with these observations, an ELISA experiment to assess the expression of several inflammatory factors showed that an increase in ATF6 protein expression significantly decreased the levels of pro-inflammatory factors (TNF-α and IL-1β) in central system fluid and serum (Figure 4A, 4B, 4D, 4E), while elevating the expression level of ATF6 promoted the expression of the anti-inflammatory factor IL-10 (Figure 4C, 4F). The brain water content indicated that when the ATF6 protein level increased, the degree of cerebral edema decreased, indicating that ATF6 can alleviate cerebral edema caused by ICH (Figure 4G).

### ATF6 upregulation mitigates ICH-induced brain damage by inhibiting neuronal cell death, brain edema, preserves blood-brain barrier integrity and oxidative stress response

**Figure 5:**
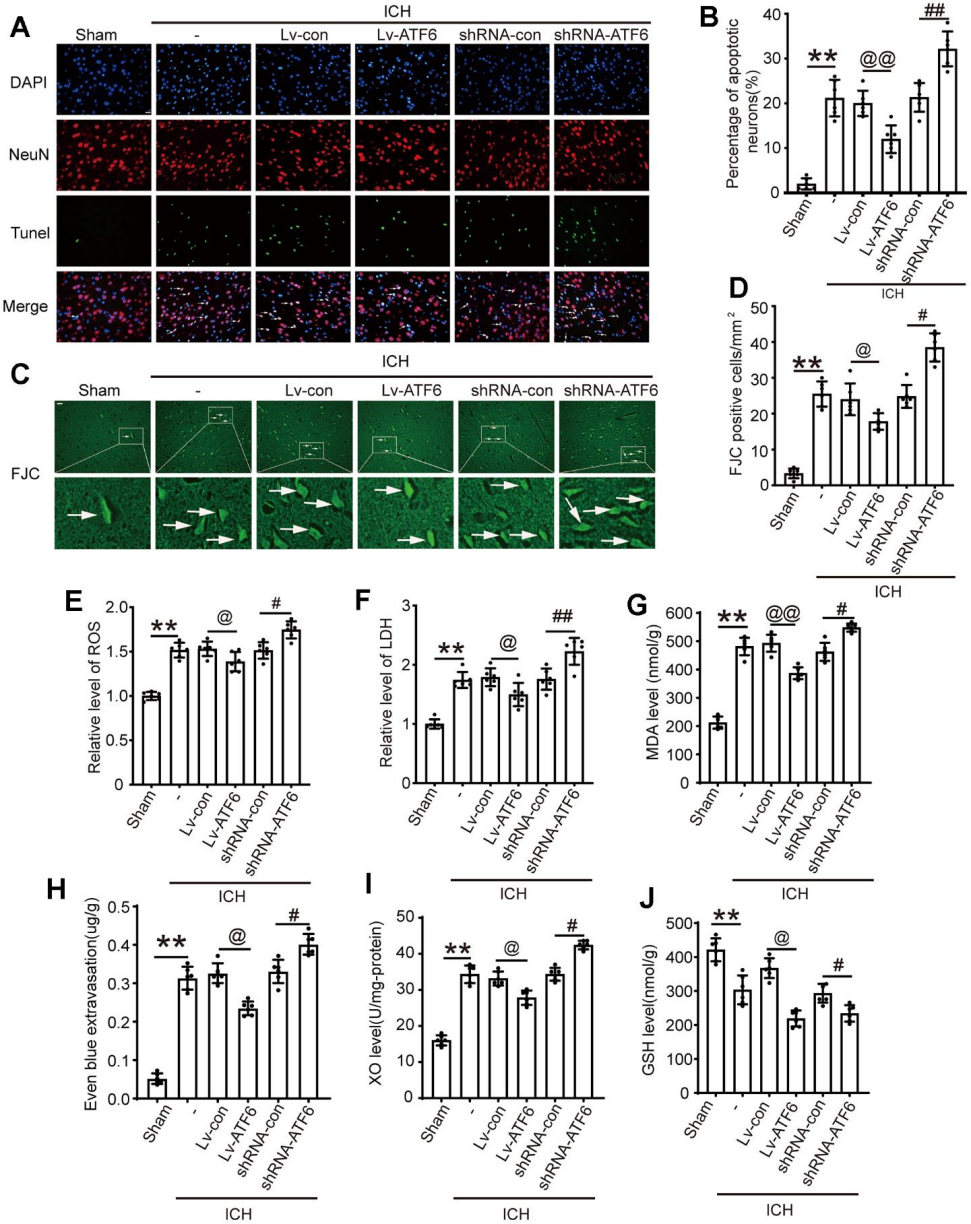
**Up-regulation of ATF6 reduced apoptosis and neuron loss induced by ICH.** (**A**, **B**) Apoptotic cells were labeled in brain sections using TUNEL staining, and the percentage of apoptotic cells was analyzed statistically. Arrow indicated TUNEL positive cells. Nuclei were labeled with DAPI (blue). (**C**, **D**) FJC staining. Arrow indicated FJC positive cells. (**E**) ROS. (**F**) LDH. (**G**) MAD. (**H**) Even blue extravasation. (**I**) XO Level. (**J**) GSH level. Scale bar = 50 um. The black dots represent individual data in each group. **p < 0.01 and *p < 0.05, ^@@^*p* < 0.01 and ^@^*p* < 0.05, ^##^*p* < 0.01 and ^#^*p* < 0.05, n = 6.

We employed TUNEL assays to assess the influence of ATF6 on neuronal apoptosis and Fluoro-Jade C (FJC) staining to investigate neurodegeneration under different interventions. The ratios of neurodegeneration and cell death markedly increased in ICH-induced brain tissues compared to those in the Sham group. TUNEL staining data revealed that apoptotic neuron numbers increased significantly after shRNA-ATF6 treatment but decreased with ATF6 over-expression (Figure 5A, 5B). Similarly, FJC staining indicated that compared with the shRNA-con group, when the expression level of ATF6 decreased, the death cells increased sharply (Figure 5B, 5D). Oxidative stress response was assessed through markers such as ROS, XO, MDA, LDH, and GSH. Our results demonstrated that the oxidative stress response in the shRNA-ATF6 group was significantly increased compared to the Lv-ATF6 group (Figure 5E–5G, 5I, 5J). Therefore, ATF6 upregulation led to a reduction in oxidative stress and cellular necrosis in ICH subjects. Similarly, by increasing the expression level of ATF6 protein, we could observe that the degree of damage to the blood-brain barrier was reduced in this group of rats, indicating that ATF6 effectively protected the brain tissue (Figure 5H).

### Impact of shRNA-ATF6 and Lv-ATF6 treatments on cognitive behavior in ICH-induced rats

**Figure 6:**
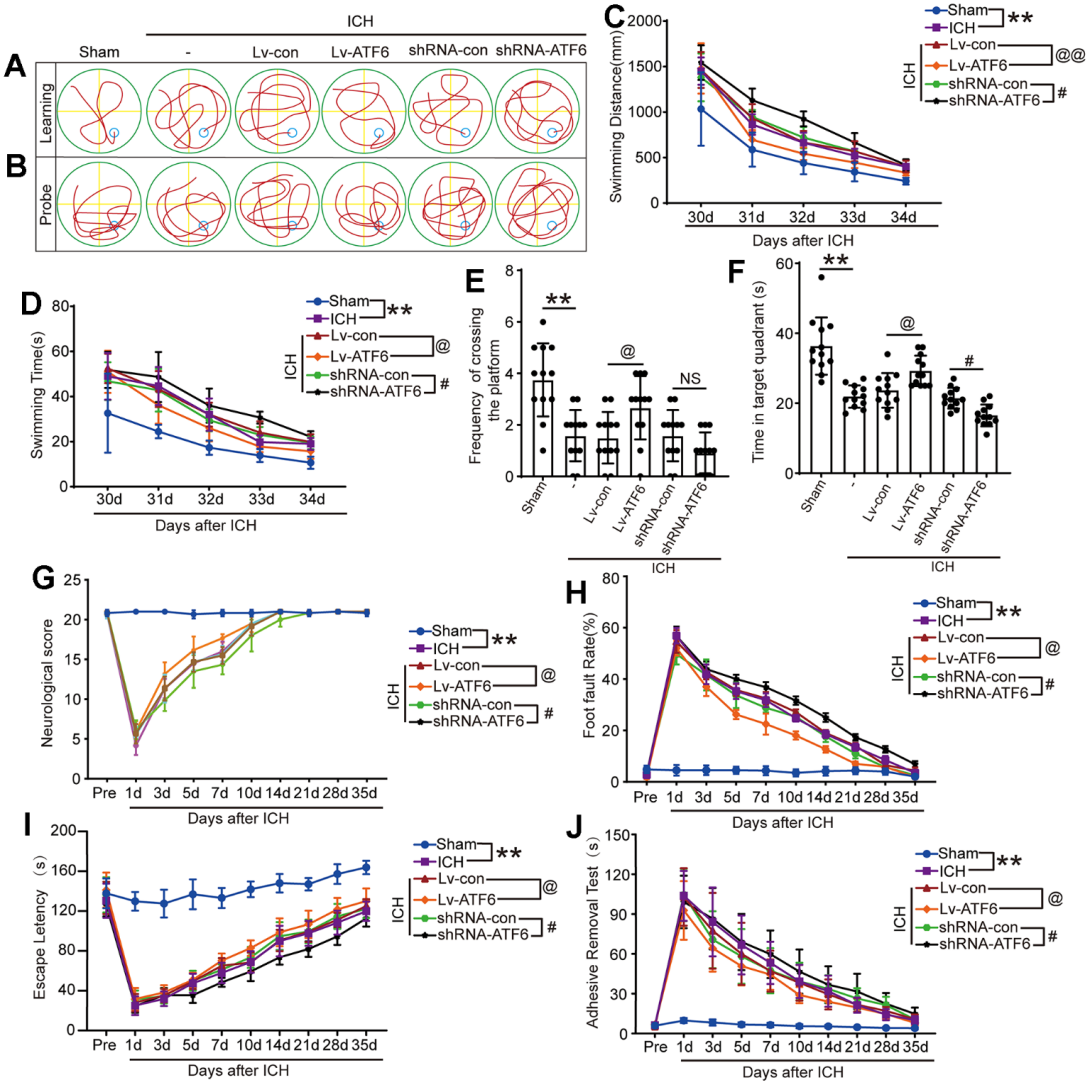
**Downregulation of ATF6 can aggravate ethology deficits after ICH.** (**A**) Representative track of Morris water maze experiment during 30-34 days after ICH. (**B**) Representative track of Morris water maze experiment on 35 days after ICH. (**C**) Swimming distance. (**D**) Swimming time. (**E**, **F**) Time in target quadrant and frequency cross the platform of Morris water maze experiment on 35 days after ICH. (**G**) Neurobehavioral score. Higher scores suggest less neurobehavioral deficits. (**H**) Foot fault test. (**I**) The motor coordination ability of rats after ICH was evaluated by rotarod test, long latency time indicates good motor coordination ability. (**J**) Adhesive-removal test. The black dots represent individual data in each group. **p < 0.01 and *p < 0.05, ^@@^*p* < 0.01 and ^@^*p* < 0.05, ^##^*p* < 0.01 and ^#^*p* < 0.05, n = 12.

To further ascertain the role of ATF6 in ICH-induced brain damage, we evaluated cognitive function and locomotor impairments using ethology test. Morris water maze experiment can study the learning and memory function of ICH animals. The results showed that, in the Lv-ATF6 group, the time to find the platform was significantly shorter than in the Lv-con group, and the swimming distance was significantly shorter than that of the Lv-con group. Reversely, in the shRNA-ATF6 group the time to find the platform was significantly longer than in the shRNA-con group, and the swimming distance was significantly increase. In terms of time and distance, there was no significant difference between the ICH, ICH + shRNA-con and ICH + Lv-con groups (Figure 6A, 6C, 6D). The groups also differed in memory, as the Lv-ATF6 group spent significantly more time in the second quadrant than the Lv-con group, and crossed the platform position more frequently than the Lv-con group, while the shRNA-con group spent more time in the second quadrant than the shRNA-ATF6 group, and crossed the platform position slightly more frequently than the shRNA-ATF6 group (Figure 6B, 6E, 6F). Neurobehavior score indicated that compared with the Lv-ATF6 group, the Lv-con group had a lower score, while the shRNA-con group had a significantly higher score than the sshRNA-ATF6 group (Figure 6G). Through foot fault, rotarod and adhesive removal experiments, we concluded that the balance, movement and sensory abilities of rats in Lv-ATF6 group were significantly better than those in Lv-con group (Figure 6H–6J).

## DISCUSSION

ICH often triggers numerous adverse effects, including neuroinflammation and apoptosis in the affected perihematomal tissues. Factors contributing to apoptosis typically include decreased blood flow and metabolism at the hematoma periphery, activation of several blood enzymes, and mechanical hematoma alteration under ICH induction [38]. Previous studies have underscored the significance of ATF6 in cell death, inflammation, and oxidative stress response [22, 39]. Moreover, evidence suggests that AFT expression is considerably enhanced in mature central and peripheral neurons following axotomy and ischemia, hinting at ATF6’s role in responding to neuronal injury [40, 41]. Our study aimed to investigate the impact of ATF6 on secondary brain injury (SBI) caused by ICH. Results revealed a substantial increase in ATF6 protein expression in brain tissues affected by ICH, and that augmenting ATF6 expression significantly ameliorated SBI caused by ICH. Therefore, ATF6 may serve a crucial neuroprotective role after ICH.

Relevant literature has reported that 3h, 12h and 24h are critical time points in the acute phase (within 24h) after intracerebral hemorrhage [42]. In this study, we selected 1h, 3h, 6h, 12h, 24h and 48h after ICH to conduct the test. Because these several times we have chosen are the most critical time points for the progression of the disease after cerebral hemorrhage. We measured the expression trend of ATF6 after ICH and found that the expression level of ATF6 gradually increased after ICH, reached its peak at 24 hours after ICH, and then began to slowly decline. Similarly, we noticed an increase in CTH expression as ATF6 levels changed. It can be inferred that CTH also plays an important role after ICH, but the specific role needs further experimental investigation. We then proceeded to explore the key role ATF6 plays after ICH. Since the expression level of ATF6 is highest at 24h after ICH, the intervention of ATF6 expression at this time point is more likely to be successful, and there are fewer other influencing factors, and the subsequent experimental results are more convincing. We first successfully regulated ATF6 expression levels using interference reagents, ensuring the reliability of subsequent experimental results. We were surprised to find that CTH changes with the expression level of ATF, which indicates that the role of ATF changes through affecting the expression level of CTH. Our experiments primarily focused on the inflammatory response. Microglia, the central nervous system’s innate immune cells, exhibit anti-inflammatory (M2) and pro-inflammatory (M1) phenotypes [43, 44]. An increase in ATF6 expression levels corresponds to an increase in M2 type Microglia. Notably, M2 type Microglia can effectively inhibit inflammatory reactions and foster brain injury recovery [45]. We thus posit that after ICH, ATF6 may promote CTH levels to induce a transformation of Microglia phenotype into M2 type, thereby inhibiting the inflammatory response and aiding nervous system damage repair. Furthermore, our ELISA experiment corroborated the staining results. An increase in ATF6 expression level was associated with a sharp increase in IL-10 expression and a decrease in pro-inflammatory factors IL-1β and TNF-α. The results of two independent experiments indicated that after ICH, ATF6 promoted the transformation of Microglia into M2 type by regulating the expression of CTH, inhibited the inflammatory reaction, and aided injury repair.

Previous research has suggested that apoptosis is a common form of cell death after ICH and a primary cause of ICH-induced brain injury [46]. Our findings indicated that ATF6 upregulation increased the quantity of surviving neurons and decreased the number of TUNEL-positive neurons in ICH-induced brain tissue. Similar results were obtained through FJC staining. This suggests that ATF6 may prevent brain cell death and potentially promote recovery of damaged neural function, providing molecular evidence for the neuroprotective effect of ATF6. When conducting the cell death experiment, we only selected the brain tissue sections of the basal ganglia for observation. We can only ensure the consistency of each group of sections through randomness, and ensure the rigor of the experiment through the repetition of 6 groups of experiments.

Apart from influencing apoptotic signaling pathways, ICH-induced brain tissue damage is also associated with brain edema, oxidative stress, and neurobehavioral dysfunction. Further investigation into the molecular effects of ATF6 revealed that augmenting ATF6 expression diminished blood-brain barrier (BBB) impairment, brain edema, and oxidative stress in ICH rats. This implies that ATF6 also extends its protective capabilities through other pathways to mitigate brain damage. The above molecular level experiments have demonstrated that ATF6 can inhibit the inflammatory response after ICH and alleviate brain injury. However, elucidating the function of ATF6 at the molecular level still lacks persuasiveness, so it is necessary to conduct short-term and long-term behavioral validation at the individual level.

The research results indicate that increasing the expression level of ATF6 can enhance the motor, learning, and perceptual abilities of rats, providing convincing evidence for the protective effect of ATF6 on the brain. In the experiment, we only explored the role of ATF6, so we designed three control groups: ICH group, shRNA con group, and Lv-con group. However, there was no statistically significant difference between these three groups of experiments, so the only variable was the change in ATF6, while the other experimental conditions were consistent, including hematoma decomposition products.

From a series of experiments, we infer that after ICH, ATF6 inhibits neuroinflammation, oxidative stress, blood-brain barrier damage, brain edema, and cell death, thereby promoting neurological function recovery. Therefore, the results of this series of behavioral experiments indicate that reducing the expression level of ATF6 contributes to the recovery of rat behavior, which is consistent with the results of molecular experiments. The overall conclusion indicates that reducing the expression of ATF6 helps to reduce inflammation in the central nervous system, reduce cell death, reduce brain edema, protect the integrity of the blood-brain barrier, and alleviate the damage of cerebral hemorrhage to the central nervous system. This is reflected at the individual level and can promote the recovery of neurological function.

Nevertheless, our study had several limitations. First, although our experiment has made some research on the brain protective effect of ATF6, we have not studied the relevant protective mechanism in depth. Therefore, in the following experiments, we will focus on the brain protective mechanism of ATF6. Second, our experiment mainly discussed the related role of ATF6 in the acute phase of ICH, investigating the long-term effects and sustained ATF6 expression beyond 48 hours could provide insights into its role in the chronic phase of ICH recovery, we will focus on this part of the experiment in the future research. Finally, the expression of ATF6 and its role in human brain damage during ICH have not been studied using clinical samples. Finally, the precise mechanism by which ATF6 protects the brain after ICH remains unclear.

## CONCLUSIONS

Our experimental data demonstrated that ATF6 exerted a significant effect in inhibiting ICH-induced brain injury by increasing the expression level of CTH. Upregulation of ATF6 levels successfully ameliorated inflammation, neuronal apoptosis, brain edema, BBB injury, and neurological dysfunction. Hence, these findings may help to guide the future clinical treatment of SBI after ICH.
